# Spearmint targets microtubules by (−)-carvone

**DOI:** 10.1093/hr/uhae151

**Published:** 2024-05-28

**Authors:** Nathalie Hering, Anne-Catherine Schmit, Etienne Herzog, Louis-Thibault Corbin, Leona Schmidt-Speicher, Ralf Ahrens, Marie-Laure Fauconnier, Peter Nick

**Affiliations:** Joseph Gottlieb Kölreuter Institute for Plant Sciences (JKIP), Karlsruhe Institute of Technology, Karlsruhe 76131, Germany; Institut de biologie moléculaire des plantes (IBMP), CNRS, Université de Strasbourg, Strasbourg 67084, France; Institut de biologie moléculaire des plantes (IBMP), CNRS, Université de Strasbourg, Strasbourg 67084, France; Institut de biologie moléculaire des plantes (IBMP), CNRS, Université de Strasbourg, Strasbourg 67084, France; Institute of Microstructure Technology (IMT), Karlsruhe Institute of Technology, Eggenstein-Leopoldshafen 76344, Germany; Institute of Microstructure Technology (IMT), Karlsruhe Institute of Technology, Eggenstein-Leopoldshafen 76344, Germany; Gembloux Agro-Bio Tech, University of Liège, Gembloux 5030, Belgium; Joseph Gottlieb Kölreuter Institute for Plant Sciences (JKIP), Karlsruhe Institute of Technology, Karlsruhe 76131, Germany

## Abstract

Allelopathy can provide sustainable alternatives to herbicides because it is based on specific signals rather than generic toxicity. We show that the allelopathic activity of Spearmint and Watermint is linked with their main compounds, (−)-carvone and (+)-menthofuran, both deriving from (−)-limonene. Germination of Poppy and Cress, and root growth of *Arabidopsis thaliana* are inhibited by very low concentrations of (−)-carvone, acting even through the gas phase. (+)-Menthofuran is active as well, but at lower efficacy. Using fluorescently tagged marker lines in tobacco BY-2 cells and *Arabidopsis* roots, we demonstrate a rapid degradation of microtubules and a remodeling of actin filaments in response to (−)-carvone and, to a milder extent, to (+)-menthofuran. This cytoskeletal response is followed by cell death. By means of a Root Chip system, we can follow the tissue dependent response of the cytoskeleton and show a cell-type dependent gradient of sensitivity between meristem and distal elongation zone, accompanied by programmed cell death.

## Introduction

Allelopathic phenomena were firstly described by Hans Molisch [1], who conceptualized them on a more general level as manifestations of chemical communication between plants mediated by organic molecules, called allelochemicals [2, 3]. As already pointed out by Hans Molisch [1], many allelochemicals exert their effect over a distance, which means that they are often volatile compounds. Thus, by their chemical nature, they connect hitherto separated individuals by synergistic or antagonistic interactions, establishing a system (*in sensu* [4]) extending beyond the realm of the individual plant.

A classic example are volatiles released by Sagebrush in consequence of herbivory that will induce herbivore defense in the neighboring plants, even across species borders, an effect that can be partially ascribed to methylated jasmonate formed in consequence of wounding, but also to volatile sesquiterpenes [5]. In general, terpenoids account for a substantial fraction of plant volatiles. For instance, conifers can emit up to 2% of their dry weight as terpenes, especially in response to stress [6]. While some volatiles can act as signals between individuals, one has to keep in mind that these volatiles can also be used as systemic signals within the individual plant itself. For instance, isoprene, the building block of terpenoids, has been shown to increase thermotolerance of leaves [7]. Likewise, in Rice, oxidative stress caused, for instance, by UV-B irradiation, can induce a terpene synthase culminating in the production of the monoterpene (S)-limonene that buffers the challenged photosystem against reactive oxygen species [8]. This example indicates a scenario, where systemic signals, originally used to orchestrate the response of the individual plant to stress, were later used to steer the allelochemical interaction between different individuals.

Essential oils of the Lamiaceae are very rich in such monoterpenes [9], and these compounds are also underlying the human use of these plants for medicinal, cosmetic, and culinary purposes [10, 11]. The Lamiaceae are also known as extremely diverse, comprising 7200 species in 236 genera [12], including the economically relevant genus *Mentha* that accumulates specific, value-giving compounds [13]. The different Mints are all endowed with a characteristic and individual scent, meaning that they all strongly differ in their chemical profiles [14–16]. This chemical diversity stimulates the following questions: Why has evolution driven Mints to unfold this degree of chemical variation? Is this a sign of specificity? In biology, specificity is often associated with signal transduction. So, can these chemical compounds be understood as specific signals, or are they just compounds with a generic bioactivity, for instance insect repellence or phytotoxicity?

It is well known that essential oils from Mints can exhibit a high bioactivity, for instance, against bacteria, fungi, or even viruses [17]. Also, allelopathic activities have been reported. For instance, essential oil of *M. spicata*, which is rich in (−)-carvone, could almost eliminate the germination of the pertinent weed *Amaranthus retroflexus*, while half of the seeds of the ornamental plant *Alcea pallida* were still germinating, demonstrating that suppression depends on the recipient species [18] and is unlikely to be caused by an overall phytotoxicity. This specificity is in sharp contrast with the generic effect of synthetic herbicides and has also been found for other essential oils, such as that of *Eucalyptus* [19] or Horsemint [20].

The cellular mode of action underlying this specificity is, however, still poorly understood. Microtubules appear to be a significant target for various classes of secondary compounds due to their involvement in diverse cellular processes such as mitosis and cell expansion, which are crucial for plant development and morphogenesis [21]. For example, treatment of *Arabidopsis* roots with the phenylpropanoid scopoletin resulted in pronounced cell and tissue abnormalities, likely due to the reorganization of microtubules from a transversal to longitudinal orientation, thus leading to cell death, reminiscent of the effect of auxin herbicides [22]. Another phenylpropanoid, gallic acid, has been demonstrated to disrupt microtubules, probably through the accumulation of reactive oxygen species (ROS), leading to the perturbation of root architecture in *Arabidopsis* [23]. In addition, a further class of secondary compounds interferes with the microtubules. The alkaloid norharman has been shown to inhibit root growth in *Arabidopsis* by disrupting the arrangement of the microtubules and ultimately leading to cell death [24]. Some terpenoids also act on microtubules. The sesquiterpene farnesene inhibited root growth in a dose-dependent manner, altered the structure and organization of root cells, and led to left-handed growth due to a loss of perception of gravitropic stimuli, which is attributed to microtubule disorganization and hormonal imbalance in *Arabidopsis* [25]. In a previous study [26], we could identify the monoterpene menthone as active compound for the allelopathic effect of Korean Mint. Here, menthone was able to disrupt microtubules in tobacco cells expressing a fluorescent tubulin reporter, whereas menthol, which differs from menthone only by a hydroxyl- instead of a keto-group, required a tenfold higher concentration to achieve the same degree of microtubule elimination. Likewise, the mixture of the acyclic monoterpenes geranial and neral, namely citral, was shown to target microtubules in *Arabidopsis* seedlings in a dose-dependent manner [27]. This study was extended in 2012 by Chaimovitsh *et al.*, where they showed that citral causes significant inhibition of root elongation depending on dose and time in wheat seedlings. They demonstrated that citral interferes with mitotic microtubules, causing cell cycle arrest as well as deformed cell plates [28]. Microtubules have been an interesting target for synthetic herbicides as well, because they are not only the building blocks for the division spindle, but also determine the axiality of cell growth through orienting cellulose deposition (for review see [29]). When microtubules in the distal elongation zone of the root get disrupted by chemical compounds, such as colchicine, the cells stop elongating and begin to expand laterally, leading to a characteristic bulging [30]. This mode of action is exploited for several classes of herbicides, including the dinitroanilines, phosphoric amides, and the N-phenyl carbamates [31]. However, these compounds do not allow for specificity, because tubulins are conserved proteins and, thus, anti-microtubular compounds are not specific for weeds, but cause collateral damage to other species that are not in the scope of the management strategy.

The fact that a monoterpene such as menthone or a mixture of acyclic monoterpenes such as citral acts against microtubules while others, such as menthol, do not show that the bioactivity of these compounds must be of a completely different nature. This supports a model, where the specificity of the effect is caused by altered signaling in the recipient plant. A signal requires a receptor, which can be operationalized by two properties. First, the receptor must have a specific binding site for a primary signaling molecule. Second, it must convey a downstream signal by itself. Monoterpene receptors are known for animals, such as the menthol receptor TRPM8 that, at the same time, triggers a sensation of chilling [32]. However, while distant homologues of this receptor can be found in different clades of the metazoa, they seem to be absent from plants. Nevertheless, recently, the receptor for the sesquiterpene β-caryophyllene could be identified as TOPLESS [33]. This transcriptional co-repressor can be recruited to a complex with the jasmonate receptor JAZ and mediates the suppression of jasmonate-responsive genes. Binding of the active hormone jasmonate-isoleucine causes proteolytic decay of JAZ, releasing TOPLESS and, thus, relieving the inhibition of transcription [34]. The reversible acetylation of TOPLESS can regulate jasmonate signaling [35], and β-caryophyllene, the ligand of TOPLESS, can indeed activate herbivore defense, depending on jasmonate [36]. Whether volatiles other than β-caryophyllene can be sensed in a similar manner, has not been investigated so far. Since TOPLESS is organized as a gene family, which is part of the extensive Groucho/Tup1 group of co-repressors (for review see [37]), co-receptor diversity might well account for the observed specificity of plant responses to volatile plant compounds.

In the current study, we focus on two Mint species with significant allelopathic activity, namely Spearmint (*Mentha spicata*) and Watermint (*Mentha aquatica*). The germination suppression of their essential oils could be attributed to the main compounds (−)-carvone (Spearmint) and (+)-menthofuran (Watermint). Especially (−)-carvone was efficient in inhibiting germination of Cress and Poppy at low concentration, while for (+)-menthofuran higher concentrations were required to reach a similar inhibition. Using fluorescently tagged marker lines in tobacco BY-2 cells as well as roots of *Arabidopsis*, we show that both compounds eliminate microtubules and cause a bundling of actin filaments, which is followed by cell death. Again, (−)-carvone was more efficient as compared to (+)-menthofuran. By means of a Root Chip system, we can demonstrate tissue-specificity of the microtubule response, and an apicobasal gradient of this response, initiating in the meristem and progressing into the distal elongation zone. The specific effect on the cytoskeleton renders (−)-carvone a promising candidate for the development of a specific bioherbicide.

## Results

### The profiles of essential oils are species specific, in a non-additive manner

**Figure 1:**
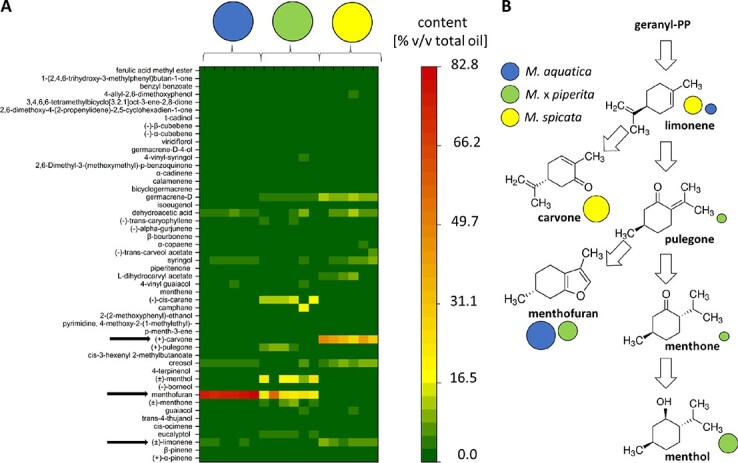
Differential composition of essential oil in three closely related *Mentha* species via GC–MS. Blue circles represent the parental species *M. aquatica*, yellow circles the parental species *M. spicata* var*. crispa*, green circles their natural hybrid, *M. x piperita*. (**A)** Relative contents are given as % v/v of the extracted oil. The compounds detected via GC–MS are ordered according to their retention time from bottom to top. Six biological replicates for each species were analyzed. Arrows indicate carvone, menthofuran, and limonene. (**B)** Pathway of the main components. Relative abundance is shown by the different size of the circles representing the different species.

Since the chemical profiles depend on the environmental conditions, the development, and also the chemotype, we have carried out a chemical analysis of the oil composition. As first step, we searched for species-dependent differences in the composition of essential oils between the parental species, Spearmint (*M. spicata* var. *crispa*) and Watermint (*M. aquatica*), as well as their natural hybrid, Peppermint (*Mentha x piperita*). Essential oils from six individuals per species were extracted by hydro-distillation and analyzed via GC–MS (Fig. 1A). The profiles are represented according to retention time from bottom to top and the proportion of the identified compound as percentage of the total oil is given by a color code. Although the three species are phylogenetically closely related, they are chemically very different (Fig. 1B). In Watermint (*M. aquatica*), menthofuran was very clearly the main component, accounting for about 80% of the essential oil. In contrast, the immediate precursor pulegone was not detectable, while limonene, an early precursor, was present, although in low amounts. In the descendant, Peppermint (*M. x piperita*), menthofuran was still the main component, albeit at a far lower abundance (about 25%) and accompanied by almost the same level of menthol (about 20%), and lower (5–10%) amounts of its precursor menthone. Here, the precursor pulegone was detectable at around 5–10% abundance, while the early precursor, limonene, was not. In contrast, the essential oil of Spearmint (*M. spicata* var. *crispa*) differed strongly from that of the other two species. Here, carvone was the predominant component with a relative content of about 35–45%. Additional signatures were the presence of creosol (up 5–10%) in Spearmint, and cis-carane in Peppermint (10–20%). Spearmint oil contained, in addition, small amounts (around 5–10%) of germacrene D and dehydroacetic acid. Overall, the chemical profiles of the three species are specific, and the chemical composition of Peppermint oil cannot be understood as mere additions of the compounds present in the parental species Watermint and Spearmint. On the one hand, new compounds (such as menthol or cis-carane) accumulate in Peppermint that are not seen in any of the parental species, while on the other hand, compounds that are abundant in one of the parents are either absent (carvone, dominant in Spearmint), or strongly depleted (menthofuran, dominant in Watermint) in the descendant, Peppermint.

### (−)-Carvone and (+)-menthofuran specifically inhibit germination of cress and poppy

**Figure 2:**
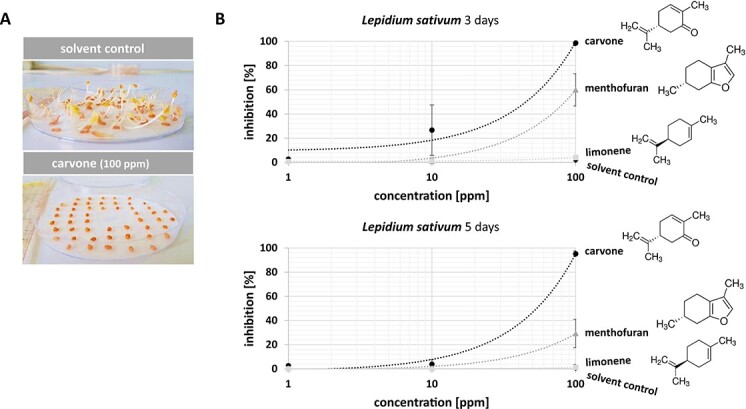
(−)-Carvone inhibits germination of Cress. (**A)** Representative image of the phenomenon. The upper image shows the outcome after applying the solvent control *n*-hexane, the lower image after application of 100 ppm (−)-carvone, both administered through the gas phase. (**B)** Dose–response of germination inhibition over the concentration of (−)-carvone as compared to the structurally related monoterpenes (+)-menthofuran and (−)-limonene, as well as compared to the solvent control after incubation in darkness at 25°C for either 3 or for 5 days. A linear trend line is indicated. Data represent means and SD from biological triplicates.

Since our previous work [20, 26] has shown specific allelopathic activities for essential oils from different Mint species, we assessed a potential bioactivity of (−)-carvone, the main component of Spearmint, and (+)-menthofuran, the main component of Watermint, in a standard Cress germination assay (Fig. 2). For the assay, 50 seeds were sown on a moistened Whatman filter paper and the inhibition rate was quantified after 3 and 5 days. Already after 3 days, a dose-dependent inhibition of germination became detectable in response to an interaction with (−)-carvone through the gas phase. Exposure of Cress seed to the highest tested concentration of (−)-carvone (100 ppm) produced an almost complete inhibition of germination. This inhibition was persistent, those seeds that were inhibited at day 3 (almost 95%), remained inhibited, when scored at day 5. When the concentration was reduced to 10 ppm, the inhibition was weaker, but still significant with around 25% at day 3. Interestingly, this weaker inhibition was less persistent, because at day 5, the value had dropped to around 5%, which means that the majority of the seeds inhibited at day 3, were able to resume germination afterwards. Thus, both with respect to the amplitude of inhibition, as well as with respect to its persistence, the effect of (−)-carvone was dose-dependent. The bioactivity of (+)-menthofuran, although detectable, was much weaker. Here, the inhibition reached at day 3 was only 60%. Moreover, this inhibition was not persistent, because at day 5, the inhibition had dropped to around 30%, meaning that around half of the seeds that had been initially suppressed, could enter germination later. The solvent for both compounds, *n*-hexane, had no effect on germination whatsoever. Also, (−)-limonene, the common precursor for both, (−)-carvone and (+)-menthofuran, failed to produce any significant inhibition, and was, therefore, not pursued further in the subsequent assays.

**Figure 3:**
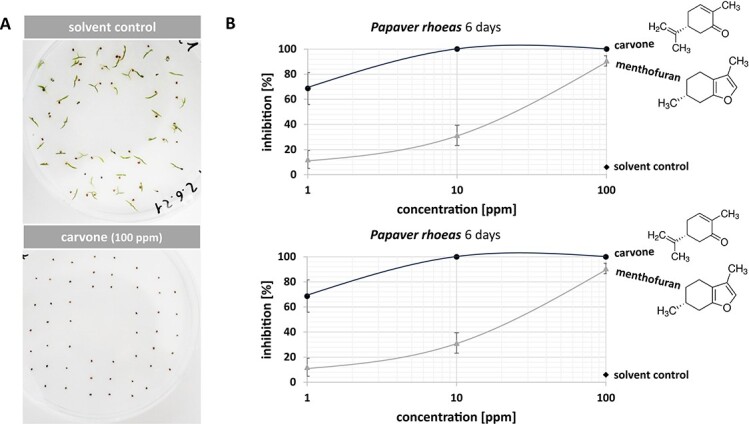
(−)-Carvone inhibits germination of Poppy. **(A)** Representative image of the phenomenon. The upper image shows the outcome after applying the solvent control *n*-hexane, the lower image after application of 100 ppm (−)-carvone, both administered through the gas phase. (**B)** Dose–response of germination inhibition over the concentration of (−)-carvone as compared to the structurally related monoterpene (+)-menthofuran, as well as compared to the solvent control after incubation in a 12 h light/12 h dark cycle at 20°C for 2 or 6 days. A saturation curve is indicated. Data represent means and SD from biological triplicates.

To assess, whether the bioactivity of these monoterpenes is confined to Cress, we applied the same strategy to Poppy (*Papaver rhoeas*), one of the most pertinent weeds in winter cereals [38]. The experimental set-up was maintained, with the only difference that the experiment had to be conducted in the light, as to break the pronounced dormancy of Poppy seeds. Again, (−)-carvone was very efficient, even more efficient than in Cress (Fig. 3). Poppy responds to (−)-carvone at much lower concentrations. Already for 1 ppm, the inhibition at day 2 was almost 70%, and already for 10 ppm, the inhibition was complete. As seen already in Cress, (+)-menthofuran requires higher concentrations to inhibit germination. The shift in sensitivity compared to (−)-carvone is almost two orders of magnitude, because even at 100 ppm of (+)-menthofuran, the inhibition (around 90%) was not complete, albeit higher than for 1 ppm of (−)-carvone. For the solvent control, no inhibition could be detected. In contrast to Cress, the effect of (−)-carvone and (+)-menthofuran was persistent throughout. The inhibitions seen at day 6 were exactly the same as those at day 2. There was not a single seed that germinated later that had remained suppressed at day 2 but managed to germinate thereafter.

Summarizing, (−)-carvone yields a strong and dose-dependent inhibition of germination in both models, Cress, and Poppy. (+)-Menthofuran is active as well, but less efficiently, such that higher doses are needed to achieve the same effect. Both compounds are more efficient and more persistent in Poppy as compared to Cress. Thus, the inhibition of germination is not only depending on dose but is specific as well. The specificity becomes manifest both, with respect to the molecular details of the monoterpenes, as well as with respect to the target species.

### Dose-dependent inhibition of root growth by (−)-carvone and (+)-menthofuran.

**Figure 4:**
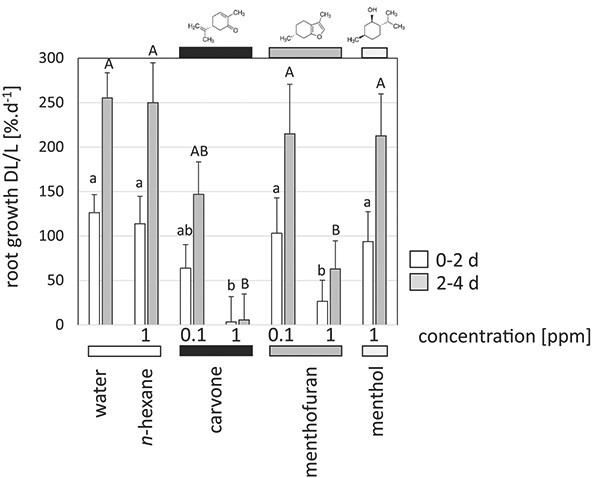
(−)-Carvone and (+)-menthofuran inhibit root growth in *Arabidopsis thaliana*. Relative growth rates (DL/L in %^.^d^−1^) during the first 2 days (0–2 d, white bars) and the subsequent 2 days (2–4 d, grey bars) of exposure to the respective compound mixed into the agar. Time zero is defined as 2 days of stratification in the dark and in the cold followed by 5 days of germination. Data represent means and standard errors from two independent series of experiments (separate Petri dishes) with 15–22 individual roots per Petri dish. Differences from the water control were tested by the non-parametric Wilcox test. Significant differences are represented by different lettering, whereby ab (AB) meaning *P* < 0.05, and b (B) meaning *P* < 0.01 compared to the water control (a or A, respectively).

The criterion for germination is the emergence of the radicle. We probed, therefore, the effect of selected monoterpenes on root elongation using seedlings of *Arabidopsis thaliana* as experimental model (Fig. 4). In addition to (−)-carvone and (+)-menthofuran, found as bioactive components in the germination assays, we also included menthol as end point of the pathway feeding the synthesis of (−)-carvone and (+)-menthofuran. To ensure a continuous and persistent pool of the components, they were administered in agar containing ½ MS medium and 0.5% sucrose. This required *n*-hexane as solvent, such that a solvent control with the maximal concentration of *n*-hexane (1 ppm) was included as well as a negative control, where roots developed on the agar without any added components beyond the MS medium and sucrose (termed as water control). Root growth was recorded from 1 week after sowing, corresponding to day 5 after germination. At this stage, root growth was accelerating—in the water control, roots more than doubled in length per day in the first 2 days of the experiment (days 5–7 postgermination), which was even increasing during the subsequent 2 days (days 7–9 postgermination), when they more than tripled in length per day. There was no change whatsoever, when roots were grown on the solvent control (1 ppm of *n*-hexane). In contrast, already 0.1 ppm of (−)-carvone yielded a significant inhibition (by around 50% compared to the water control) of root growth, and 1 ppm of (−)-carvone brought root growth to a complete halt. Also, (+)-menthofuran was inhibiting, although the effect was too mild to become significant at 0.1 ppm, while at 1 ppm a significant inhibition by around 80% as compared to the water control was observed. The end product of the pathway, menthol, was only causing a mild inhibition by around 20% compared to the water control, although applied at a concentration as high as 1 ppm. This inhibition did not cross the threshold for statistical significance, though.

Thus, the bioactivity patterns of (−)-carvone and (+)-menthofuran, seen before in the germination tests, can be recapitulated in the context of root growth: (−)-carvone is more efficient than (+)-menthofuran, the effect is dose-dependent, and it is strongly dependent on specific chemical groups, because neither the precursor (−)-limonene (germination assay), nor the end product menthol (root growth) produced any significant bioactivity.

### The cytotoxic effect of (−)-carvone and (+)-menthofuran depends on the cytoskeleton

**Figure 5:**
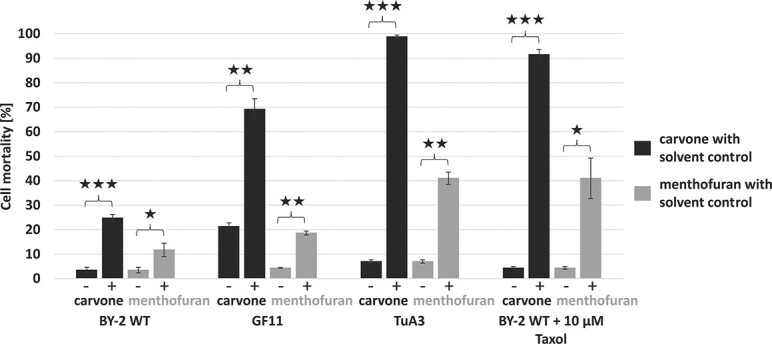
Cytotoxicity of (−)-carvone and (+)-menthofuran. Cell mortality in response to (−)-carvone and (+)-menthofuran was measured in non-transformed BY-2 cells (WT) with and without 10 μM Taxol, a specific inhibitor of microtubule disassembly, as well as in BY-2 cells expressing the actin-binding domain 2 of fimbrin fused to GFP (GF11) and cells expressing tubulin α3 from tobacco in fusion with GFP (TuA3). The values for the solvent control, *n*-hexane, are added and marked by -, while + means the respective compound (using a concentration of 0.5% (v/v)). In this experiment, the compounds were mixed with the cell suspension. Mortality was scored 30 min after addition of the compound. Data represent means and standard errors from three independent biological replications with a population of 2500 individual cells per replication. The significance was tested by a one-tailed paired t-test with ^*^  *P* < 0.05, ^**^  *P* < 0.01, and ^***^  *P* < 0.001 and indicated by asterisks.

To further dissect the mode of action of the two bioactive components, (−)-carvone and (+)-menthofuran, an Evans Blue Dye Exclusion Assay according to Gaff and Okong'o-Ogola [39] was conducted in tobacco BY-2 cells (Fig. 5). The compounds had been active with respect to the inhibition of root growth, a process, strongly dependent on the cytoskeleton. We, therefore, assessed, in addition to the wild type, cells where the cytoskeleton was labeled by green fluorescent protein (GFP)-tagged tubulin (microtubules) or a GFP-tagged domain of plant fimbrin (actin filaments). In fact, treatment with the bioactive compounds caused significant increases of mortality in all cell lines as compared to the solvent control. The cytotoxicity was much more pronounced for (−)-carvone, while the effect of (+)-menthofuran was milder, although significant. Strikingly, the strongest mortality was observed in the cell line overexpressing the fusion of GFP with α-tubulin with a mortality of almost 100%, while mortality in the actin marker line was significantly lower (around 70%), and for the non-transformed wild type even lower (around 25%). While (+)-menthofuran was less active, the sensitivity pattern of the three cell lines was similar. Here, the tubulin overexpressor line showed around 40% mortality, the actin-marker line around 19%, and the wild type only 12%. Thus, both compounds induce a significant cytotoxicity. To confirm that cell death in response to (−)-carvone and (+)-menthofuran is dependent on the presence of microtubules, it should be possible to phenocopy the phenotype of the tubulin overexpression line TuA3 in the wild type by stabilizing the microtubules with Taxol, a specific inhibitor of microtubule disassembly. In fact, pretreatment with Taxol renders the wild type more sensitive, leading to similar mortalities as seen in the tubulin overexpression line TuA3. Furthermore, the solvent control of (−)-carvone was slightly increased in the GF11 cell line. Therefore, a complementary approach measuring viability rather than mortality was performed using fluorescence diacetate assay (FDA) (Fig. S1). Here, controls showed a viability of up to 100%, while (−)-carvone caused a strong decrease in viability down to 33% and (+)-menthofuran affected the cells less strongly and they showed a viability of 76%. This mirrors the pattern observed for cell mortality using the Evans Blue Dye Exclusion Assay and confirms that there is a strong and significant difference between (−)-carvone and the solvent control regardless of the method used.

Congruent with the inhibition of germination, (−)-carvone shows a stronger bioactivity as compared to (+)-menthofuran, although (+)-menthofuran as well can exert a significant effect. The cytotoxicity of both compounds exhibits a pronounced synergy with a higher expression of tubulin (due to the additional GFP-tubulin), but also with the expression of the actin-binding domain of plant fimbrin. The effect of the two compounds is, therefore, dependent on the cytoskeleton, mainly on tubulin.

### (−)-Carvone and (+)-menthofuran rapidly degrade the cytoskeleton

**Figure 6:**
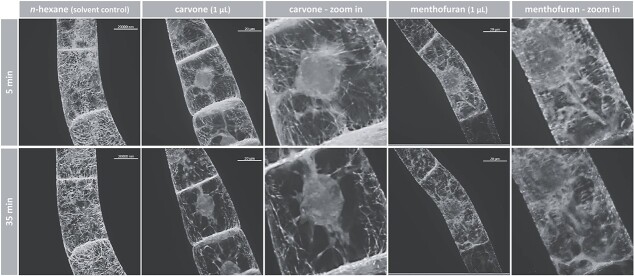
Response of microtubules to (−)-carvone and (+)-menthofuran. Representative BY-2 cells expressing tubulin α3 in fusion with GFP followed by spinning disc confocal microscopy after treatment with either 1 μL of *n*-hexane as solvent control, (−)-carvone or (+)-menthofuran at proliferation phase, day 3 after subcultivation. Maximal intensity projections of confocal z-stacks are shown. Compounds were administered through the gas phase. The volume of the solvent control, *n*-hexane, was identical (1 μL). The volume of the cell suspension was 20 μL in all experiments.

Since the mortality evoked by (−)-carvone and (+)-menthofuran was elevated in the cell lines overexpressing GFP fusions of cytoskeletal compounds, we followed the effects on the cytoskeleton in response to these compounds *in vivo* by spinning disc confocal microscopy. The components were administered through the gas phase. In the following, we avoid the term "microtubule disruption", which is commonly used but does not apply to many compounds where this has been studied, as only in very rare cases are existing microtubules actually cut into fragments. Instead, in most cases, they are eliminated by blocking the attachment of tubulin heterodimers to the plus end of the microtubules. Therefore, we introduce two definitions that describe the two mechanisms we observed: (1) Active elimination, in which the exit of dimers is promoted (2) Dimer sequestration as a mechanism by which tubulin heterodimers are prevented from being integrated into the plus-end so that the microtubule disappears due to its innate turnover. In the solvent control (1 μL of *n*-hexane), microtubules were organized in cortical arrays subtending the cell membrane (Fig. 6, left). Since the cells were viewed at day 3 after subcultivation, they were mostly in the G_2_ phase, such microtubules showed a mixed orientation, some perpendicular with the axis of the cell file, some oriented deviantly. The first time point was set to 5 min after exposure of the cells to the compounds, because adjustment, focusing, and selection of suitable cells required around 1–3 min. Although individual microtubules showed certain dynamics with respect to orientation, the solvent did not cause any significant effect over the entire 30 min of the time series. This was in sharp contrast with the situation after exposure to (−)-carvone. Here, already after 5 min, the microtubular arrays appeared actively eliminated, and 30 min later only a few residual perinuclear rods and scarce longitudinal microtubules in the cortex remained (Fig. 6, center). For (+)-menthofuran, microtubules were affected as well, but to a lesser extent and with a different pattern (Fig. 6, right). Here, the partial elimination of microtubules was accompanied by the accumulation of a diffuse cytoplasmic signal, presumably originating from sequestered and disassembled tubulin dimers.

**Figure 7:**
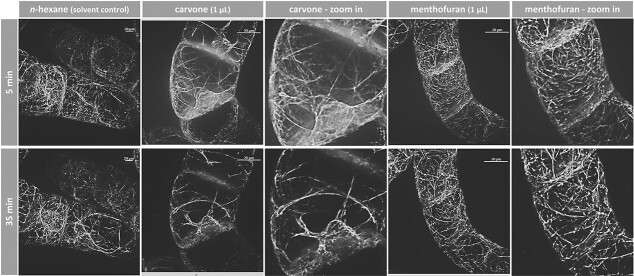
Response of actin filaments to (−)-carvone and (+)-menthofuran. Representative BY-2 cells expressing the actin-binding domain 2 of fimbrin in fusion with GFP followed by spinning disc confocal microscopy after treatment with either 1 μL of *n*-hexane as solvent control, (−)-carvone or (+)-menthofuran at proliferation phase, day 3 after subcultivation. Maximal intensity projections of confocal z-stacks are shown. Compounds were administered through the gas phase. The volume of the solvent control, *n*-hexane, was identical (1 μL). The volume of the cell suspension was 20 μL in all experiments.

In the next step, we investigated the response of actin filaments to (−)-carvone and (+)-menthofuran. Again, the actin cytoskeleton maintained its integrity over time, when exposed to the solvent, *n*-hexane (Fig. 7, left). On the contrary, (−)-carvone induced a rapid and massive bundling of actin cables and a contraction on the nucleus (Fig. 7, center), an effect, clearly seen already at the first time point, 5 min after exposure to (−)-carvone. For (+)-menthofuran, there was only a very weak impact. Even at the end of the experiment, after 35 min, only a slight bundling was detected if compared to the initial time point.

In summary, the activity pattern for (−)-carvone versus (+)-menthofuran seen for the inhibition of germination (Fig. 2 and 3), root-growth (Fig. 4), and the induction of cellular mortality (Fig. 5) are reflected in the activity pattern for cytoskeletal remodeling (Figs 6 and 7). Here, (−)-carvone has an extremely strong effect on the cytoskeleton and leads to the rapid and thorough active elimination of microtubules as well as strong actin bundling. In contrast, (+)-menthofuran impacts microtubules only partially (and possibly by another mechanism, not by active elimination but by dimer sequestration) and impacts actin filaments only mildly.

### The Root Chip allows to follow microtubule responses *in planta*

The strong effect of (−)-carvone on microtubules leads to the working hypothesis that the inhibition of germination and root growth by (−)-carvone might be caused with degradation of microtubules in the growing root tip, either by blocking mitotic activity in the meristem, or by impairing cell elongation in the distal elongation zone. To check this, the Root Chip was used to follow the cellular mode of action in *Arabidopsis* roots where microtubules were tagged by AtTUB6-GFP by laser scanning confocal microscopy. To visualize cell walls and cell death, roots were counterstained with propidium iodide (at 2 μg^.^mL^−1^). This membrane-impermeable fluorescent dye binds pectins [40] and, thus, stains the cell wall. When membrane integrity becomes impaired in consequence of cell death, the dye can enter and bind to the DNA in the nucleus.

**Figure 8:**
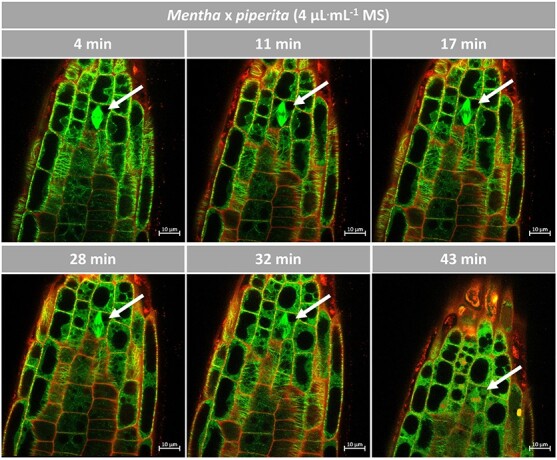
Response of microtubules to the essential oil of *M. x piperita* in *Arabidopsis*. Representative *Arabidopsis* roots expressing TuB6-GFP followed by laser scanning confocal microscopy after treatment with 4 μL^.^mL^−*1*^ of essential oil of *M. x piperita* at day 7 postgermination. Compounds were administered through ½ MS medium containing 0.5% sucrose. Propidium iodide was added to 2 μg^.^mL^−*1*^ to label the cell walls and follow cell death. The images are individual confocal sections. Observations were conducted in the multi-tracking mode using 488 and 555 nm laser excitations.

Microtubules are the targets of our investigated compounds, which showed a very high cell death rate in BY-2 cells overexpressing tubulin. Here, we also used a tubulin overexpression line from *Arabidopsis*, AtTUB6-GFP. Thus, we assumed that our compounds would also disrupt the microtubules very quickly and that this would not give us any insight into the effect on microtubules. We therefore tested the essential oil of *M. x piperita*, which was investigated in a previous study, and which showed only low bioactivity in the cress germination assay [26]. However, since the cytoskeletal response might be more sensitive than the final measured value, growth, we took the oil at a high concentration of 4 μL^.^mL^−1^ as a proof-of-concept of the Root Chip to investigate microtubule degradation and furthermore the effect on mitosis over time, which is shown in Fig. 8 and Fig. S2, where a zoom-in of the observed mitotic cell can be viewed.

In fact, by means of the Root Chip it was possible to follow the progressive degradation of microtubules and the accompanying cell death over time (Fig. 8 and S2). Here, interesting details became manifest: In the meristem, microtubules were dismantled rapidly, from around 10 min leading to a diffuse background signal in the cytoplasm (presumably from GFP-tagged tubulin heterodimers). Mitotic spindles narrowed and elongated, while individual microtubule bundles frayed out from the periphery, before, from around half an hour, the spindle lost integrity. Interestingly, the cortical microtubules in the distal elongation zone remained intact over most of the observation period and were affected only after 30 min. At the latest time point (at 43 min), they had vanished in the region bordering the meristem, while in the basal region still some microtubules persisted. This was accompanied by progressive cell death from around 30 min. Before, only the nuclei of lateral root-cap cells had been stained positive with propidium iodide as to be expected from a tissue undergoing regulated cell death. However, a comparison of the two frames recorded at 32 min and 43 min shows that in that time interval several cells of the meristem showed leakage of propidium iodide into the cytoplasm, and even in the distal elongation zone cells could be detected, where the nuclei were yellow, resulting from the overlap between the green GFP-tubulin signal and the red signal from propidium iodide.

In summary, although the essential oil of *M. x piperita* was not very efficient in blocking Cress germination, it was causing a rapid, severe, and specific degradation of microtubules. Here, meristematic cells were more susceptible as compared to the cells of the distal elongation zone. The mitotic spindle was impaired rapidly, especially in the periphery, accompanied by unusual elongation and later loss of spindle integrity. The microtubular response was clearly preceding cell death, both progressing from the meristem in basal direction into the distal elongation zone. Thus, the Root Chip allows to follow cellular responses *in planta*, preserving the functional context of root tissues.

### (−)-Carvone primarily targets microtubules in the root meristem

**Figure 9:**
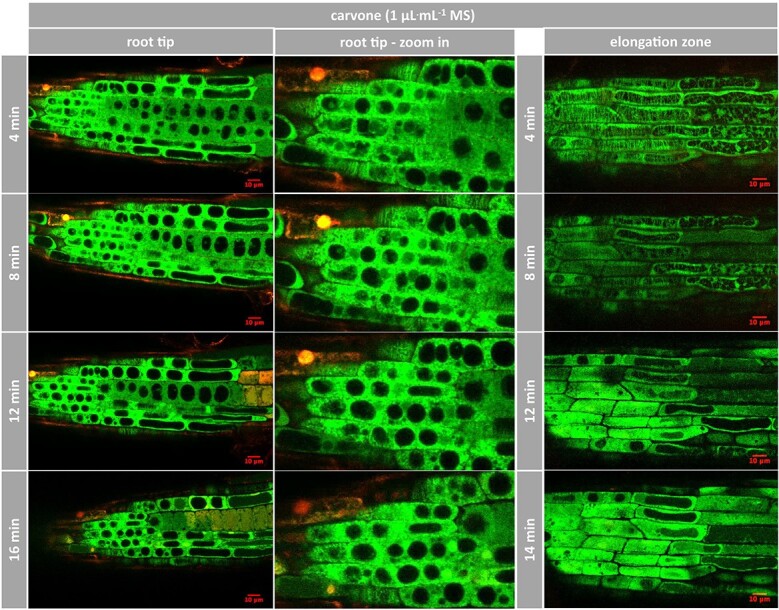
Response of microtubules to (−)-carvone in *Arabidopsis*. Representative *Arabidopsis* roots expressing TuB6-GFP followed by laser scanning confocal microscopy after treatment with 1 μL^.^mL^−*1*^ of (−)-carvone at day 7 (root tip) or day 10 (elongation zone) postgermination. Compounds were administered through ½ MS medium containing 0.5% sucrose. Propidium iodide was added to 2 μg^.^mL^−*1*^ to label the cell walls and follow cell death. The images are individual confocal sections. Observations were conducted in the multi-tracking mode using 488 and 555 nm laser excitations.

Since the Root Chip allowed valuable insights into the mode of action *in planta*, we ventured testing the effect of (−)-carvone as main compound of *M. spicata* var. *crispa* (Fig. 9). (−)-Carvone had been found to efficiently inhibit germination (Figs 2 and 3). Here, we were able to show here that the microtubules in the meristematic zone were rapidly and severely affected at a concentration of 1 μL^.^mL^−1^. Already at the first time point of 4 min (recording had a lag time, caused by handling application of the compound and re-adjustment of the optical settings), most microtubules in the meristematic zone had been replaced by a diffuse signal in the cytoplasm (Fig. 9, root tip). At the same time, cells in the elongation zone (Fig. 9, elongation zone) exhibited intact microtubules - in the distal cell (left-hand region), cortical microtubules were replaced by endoplasmic microtubules heralding the formation of root hairs. Both arrays were replaced by an intensive diffuse signal in the cytoplasm, detectable from 8 min and fully developed from 12 min. Interestingly, propidium iodide did not penetrate into those cells, suggesting that microtubules were affected at a time, when the integrity of the membrane was still intact.

**Figure 10:**
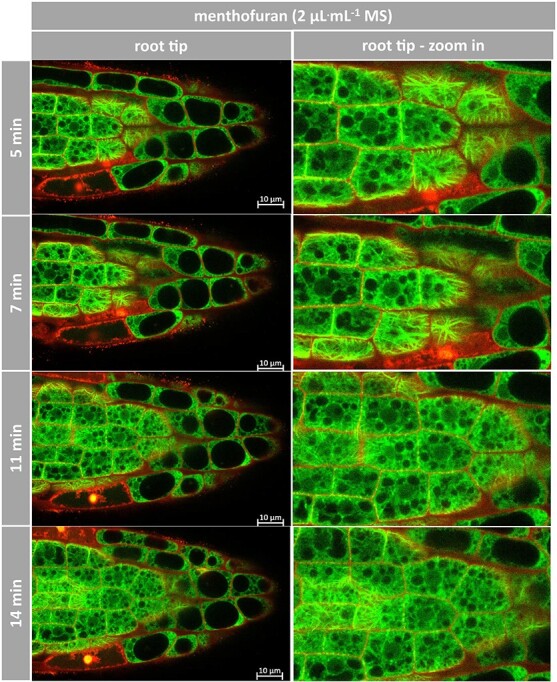
Response of microtubules to (+)-menthofuran in *Arabidopsis*. Representative *Arabidopsis* roots expressing TuB6-GFP followed by laser scanning confocal microscopy after treatment with 2 μL^.^mL^−*1*^ of (+)-menthofuran at day 7 postgermination. Compounds were administered through ½ MS medium containing 0.5% sucrose. Propidium iodide was added to 2 μg^.^mL^−*1*^ to label the cell walls and follow cell death. The images are individual confocal sections. Observations were conducted in the multi-tracking mode using 488 and 555 nm laser excitations.

Live imaging of *Arabidopsis* roots treated with (+)-menthofuran (Fig. 10) shows that this compound was not as efficient in affecting microtubules as compared to (−)-carvone. Here, even in the partially still isodiametric zone of the transition between meristem and distal, microtubules persisted over the first 10 min, and only subsequently deteriorated. Again, membrane integrity was maintained as well.

In summary, the activity pattern seen for the inhibition of germination (Figs 2 and 3), root-growth (Fig. 4), the induction of cellular mortality (Fig. 5) and cytoskeletal degradation in suspension cells (Figs 6 and 7) is reflected in the microtubular response of *Arabidopsis* roots: (−)-carvone is more efficient as compared to (+)-menthofuran. Furthermore, the Root Chip makes it possible to follow microtubular responses in the tissue context, which allows dissecting spatial and temporal patterns. The microtubular response to (−)-carvone develops in an apicobasal gradient. The meristematic cells are the most sensitive and the cells in the distal elongation zone respond later. Moreover, microtubular breakdown is probably not a unspecific consequence of cell death, as the microtubular cytoskeleton collapses, while membrane integrity still persists.

## Discussion

The current study addressed the cellular mode of action for (−)-carvone, a monoterpene with allelopathic activity from Spearmint and a comparative analysis with (+)-menthofuran from Watermint. We show that the cytotoxicity of (−)-carvone is associated with microtubule degradation and actin filament remodeling. Using a Root Chip, we also were able to address tissue specificity of the response to (−)-carvone. We observe that the microtubules in the meristem are the first to collapse, which is only later followed by cell death. These findings stimulate two main questions that will be discussed: (i) Is the inhibition of germination by (−)-carvone reflection of a generic toxicity for these compounds, or does (−)-carvone act as a signal, triggering programmed cell death of the target cell? (ii) What is the functional relevance of the microtubule response to (−)-carvone - is it part of the causal chain culminating in programmed cell death, or is it just a byproduct or even consequence of cell death?

### (−)-Carvone - a phytotoxin or a signal?

Allelopathic inhibition might be caused by a generic toxicity of the emitted compounds. There are several arguments speaking against a generic toxicity and in favor of a specific signal. First, signals act at the level of only a few molecules per cell, and the effect of (−)-carvone and (+)-menthofuran was dose-dependent at a very low concentration of 1–100 ppm. In addition, the compounds were transferred through the gas phase, so the actual concentration could be even lower to achieve an inhibitory effect. Furthermore, no physical contact with the target organism was required, in contrast to many synthetic contact herbicides (for review see [41]), where much higher concentrations are required to achieve such an effect. Second, the effect was stronger and more persistent in Poppy than in Cress. This dependence on the target plant might be due to differences in accessibility. However, the testa of Cress is far more permeable as the lignified Poppy testa, and both, (−)-carvone and (+)-menthofuran are volatile and, thus, can reach the entire interface of the seed. A third, somewhat weaker, argument is that some Cress seeds that were inhibited by (+)-menthofuran at day 3, were found to germinate at day 5, indicating that they had not been killed, but just delayed in development. This might be caused by metabolization of (+)-menthofuran possibly leading to less bioactive derivatives as found for *Arabidopsis* treated by monoterpenes [42]. In general, this implies that depending on which derivatives are produced, the allelopathic effect in the target plant can be altered or even reversed, again suggesting that monoterpenes are signaling rather than toxic compounds.

A further specificity argument comes from the pronounced difference in the monoterpene pattern between the three species. The hybrid Peppermint has inherited from the parental Watermint the ability to accumulate (+)-menthofuran, albeit to a lower extent. However, it has not inherited the ability of the parental Spearmint to produce (−)-carvone. Instead, it can generate the novel (−)-menthol, which might be linked with the lower (+)-menthofuran content, since (+)-menthofuran inhibits the expression of pulegone reductase, the key enzyme initiating the pathway towards (−)-menthol [43]. These species-specific chemical profiles are reflected by patterns of allelopathic activity that differ between the essential oils generated from Spearmint, Watermint, and Peppermint [20, 26], indicating a potential role for the ecological performance of these species.

A last, more crucial argument for a signal is the strong dependency of bioactivity on even slight chemical modifications of a compound. A single aldehyde group on the ring of (−)-carvone is linked with complete inhibition of Cress germination, while the almost identical precursor (−)-limonene, lacking just this aldehyde group, is completely inactive in this respect. Likewise, the presence of an oxygen-mediated cyclic furane moiety in (+)-menthofuran leads to growth inhibition of *Arabidopsis* roots, while the replacement of this oxygen into a hydroxylic side group in (−)-menthol leads to a complete loss of root growth inhibition. Such a strong dependence of bioactivity on molecular details were already found in previous studies. For instance, (−)-menthone from Korean Mint (*Agastache rugosa*) can disrupt microtubules, while its reduced derivative (−)-menthol fails to do so [26]. Along similar lines, citral affected microtubules, while the derivative nerol did not [27].

This dependence on specific side groups suggests that monoterpenes might bind to receptors that modulate the specific responses. For the structurally related sesquiterpene, a receptor could already be discovered [33], namely TOPLESS, a member of the extensive Groucho/Tup1 group of co-repressors (for review see [37]). Since TOPLESS is a co-repressor of jasmonate-signaling [34], binding by a ligand might promote the expression of jasmonate-responsive genes, which in some cells can activate necrotic cell death [44]. A jasmonate-dependent activity had already been shown for β-caryophyllene [36]. To what extent this signaling pathway is modulated by (−)-carvone or other monoterpenes, represents a rewarding question for future research.

### Are microtubules mediators of the death response to (−)-carvone?

(−)-Carvone caused strong dose-dependent root growth inhibition in *Arabidopsis,* similar to citral in wheat seedlings, where root elongation was significantly inhibited in a time- and dose-dependent manner due to microtubule degradation [28]. Furthermore, (−)-carvone exerted very strong cytotoxicity on BY-2 tobacco cells, especially in the transgenic TuA3 cell line, where tubulin levels are elevated [45]. Moreover, it was demonstrated that when the phenotype of the TuA3 cell line was phenocopied in the WT cell line by stabilizing the microtubules with Taxol, an inhibitor of microtubule disassembly [46], almost identical mortality rates were achieved. This suggests, on the one hand, that microtubules are involved in the programmed cell death response caused by (−)-carvone and, on the other hand, that the monoterpene-induced effect differs from an Oryzalin-type of action. If the monoterpenes directly interfere with microtubules, as Oryzalin does by sequestering tubulin dimers, overexpression of tubulin and pretreatment with Taxol should lead to a reduction in cell death instead of the observed increase, because the level of soluble dimers is increased. This indicates that an upstream signaling pathway is active that leads to microtubule degradation. To dissect the signaling pathway, further experiments will be performed with specific inhibitors or activators for components that could be upstream of microtubule degradation, e.g., GdCl_3_ to prevent Ca^2+^ influx [47] or to influence membrane fluidity using benzyl alcohol as a fluidizer [48] or DMSO as a rigidifier [49].

In BY-2 cells, the mechanisms of microtubule degradation differed between the two compounds, (−)-carvone and (+)-menthofuran, whereas they were the same in *Arabidopsis* roots. In the cells, (−)-carvone seems to actively eliminate microtubules very fast, promoting the exit of dimers (in plant interphase cells usually at the plus end, as here the minus end is closely associated with the gamma-tubulin ring complex (γ-TuRC) (for a review see [29]), as no background signal was present as with (+)-menthofuran. For (+)-menthofuran, it appears that tubulin heterodimers are prevented from being integrated into the plus-end, causing the microtubule to disappear due to its innate turnover but with a much slower and weaker antimicrotubular effect compared to (−)-carvone. However, in *Arabidopsis* roots, (−)-carvone was observed to induce a similar effect as (+)-menthofuran in BY-2 cells, namely the sequestration of dimers. This difference could be due to the presence of different microtubule-associated proteins (MAP), in particular multifunctional proteins (for review see [50]). In the roots, treatment with (−)-carvone leads to a rapid degradation of microtubules, initiating in the meristem and then progressing to the elongation zone. This contrasts with reports from a study by Chaimovitsh *et al.* [42], where microtubules in epidermal pavement cells were reported to be disrupted when exposed to (+)-carvone through the gas phase, which is already used in agriculture as an agent to suppress potato sprouting [51], but remained intact in response to (−)-carvone, the compound investigated in this study. As both studies used the same provider of compounds (Sigma-Aldrich), a scenario in which traces of the enantiomer (+)-carvone are responsible for the effect seen in the current study that were not present in the study by Chaimovitsh *et al.* [42] is not very likely. Instead, the difference between the more tightly bundled microtubules of the pavement cells, which are also protected by a cuticle, and the more dynamic and finer microtubules of the root tip, to which carvone has easy access, might well account for the discrepancy. In addition, differences in MAP, especially multifunctional ones (for review see [50]), in the various tissues could also lead to the varying sensitivity of the cytoskeleton to (−)-carvone. A further difference is the application, which in the current study, which, unlike in Chaimovitsh *et al.* [42] was not through the gas phase, but solvent facilitated through a Root Chip, expected to allow for an improved access to the target cells. However, both studies give evidence for specific signaling - in Chaimovitsh *et al.* [42] the difference in activity between the carvone enantiomers speaks against a generic toxicity, but rather for a model, where the biological effect depends on a stereospecific interaction with a binding site (in other words: with a receptor). In our study, the strong dependence of the anti-microtubular effect on specific chemical groups, as seen by the much weaker activity of (+)-menthofuran, points into the same direction. In both cases, the elimination of microtubules *in planta* is accompanied by a diffuse fluorescence in the cytoplasm, similar to the response observed in a previous study for menthone/isomenthone [26]. This diffuse fluorescence might derive from disassembled αβ-tubulin heterodimers labelled by the fused GFP. With time, this diffuse signal fades out, indicative of degradation processes. In case of the less potent (+)-menthofuran, this background persists longer as compared to the more effective (−)-carvone, where fading initiates earlier.

Since the cells begin to die soon after, the question must be posed, whether the microtubular breakdown might be the consequence of a general cellular breakdown. This aspect has been discussed earlier for (−)-menthone [26], where the loss of membrane integrity became detectable earlier than microtubular breakdown, using a dual staining with the membrane permeable Acridin Orange and the membrane impermeable Ethidium Bromide. In the current study, this question can be decided looking at the time-lapse series collected from the root tip. Here, microtubular breakdown in meristematic cells in response to (−)-carvone clearly precedes the loss of membrane integrity monitored by the entry of propidium iodide into the cytoplasm. This sequence of event is not compatible with a hypothesis, where (−)-carvone causes a breakdown of the plasma membrane and microtubules respond secondarily to this membrane breakdown. This is congruent with findings of Chaimovitsh *et al.* [42], where the loss of membrane integrity developed slower than microtubule breakdown and, in some cases, for instance for citral, microtubules were eliminated without any effect on membrane tightness. A further argument for a specific effect on microtubules comes from the fact that actin does not break down but is just massively bundled – if the primary cause of (−)-carvone were the disintegration of the membrane, this would disrupt the remodeling of actin filaments into bundles, an active process requiring phosphorylations and other post-translational modifications of actin and actin-related proteins (for a mechanistic model of this phenomenon see [52]).

What could be a possible link between a microtubular breakdown, induced by a (−)-carvone-triggered signaling event, and the observed cell death? Elimination of the microtubules is not sufficient to induce membrane permeability and, thus, cell death, so several intermediate steps must be taken. In the following, we propose a model of which steps could be interposed. Programmed cell death in plants is linked with the activation of metacaspases, the analogues of caspases that execute apoptosis in animal cells. Both caspases and metacaspases are regulated, post-translationally, by auto-processing [53]. The auto-processing of caspases depends on their interaction with MAP, such as the neural τ protein [54]. Cell death in response to microtubular breakdown is not confined to monoterpenes, such as (−)-carvone, but can also be elicited in response to phytohormonal signals, such as cytokinins [55], which lends further support to the idea that it is a signal, rather than a generic phytotoxicity, leading to the observed cell death. In fact, autophagosome components [56], or autophagosome receptors, such as Joka2 [57] were shown to be tethered to microtubules. Whether also metacaspases are regulated by tethering to microtubules, is a current topic in our research.

## Conclusion

In the current study, we demonstrate that (−)-carvone is a potent bioactive compound that causes time- and tissue-dependent degradation of microtubules in the root meristem, followed by programmed cell death that prevents root growth and, thus, unfolds allelopathic activity preventing germination of Cress and Poppy, but also of other plants, as shown in a previous study by Sarheed *et al.* [20]. The effect depends on the target species, and on the target tissue, is produced at low effective concentration, and strongly dependent on molecular peculiarities. These aspects suggest that monoterpenes act as signals rather than exerting generic phytotoxicity. So, understanding the entire signaling pathway in response to (−)-carvone at the molecular and cellular level could help us to manipulate it and develop innovative methods in agriculture, e.g. to specifically inhibit the growth of certain weeds without harming organisms outside the target groups. Thus, (−)-carvone has potential for the development of a specific bioherbicide valorizing the natural phenomenon of allelopathy, and providing plant protection alternatives that can help safeguarding biodiversity.

## Material and methods

### Plant material

The Mint and *A. thaliana* (Col 0) plants used in this study derive from the JKIP Experimental Station of the Karlsruhe Institute of Technology (KIT), Karlsruhe, Germany. Cress seeds for the germination assay were purchased from a commercial source (Rapunzel, Legau; Germany), while the Poppy seeds originated from the seed bank of the JKIP Experimental Station of the KIT. The fluorescent marker line of *Arabidopsis* GFP-TuB6 over-expressed a N-terminal fusion of β-tubulin 6 from *A. thaliana*, which was N-terminally fused with GFP under a Cauliflower Mosaic Virus (CaMV) 35S promoter generated in the Col-0 background [58] and was kindly provided by Dr. Kateřina Schwarzerová, Institute of Plant Physiology, Charles University, Prague. Details including voucher numbers are given in Table 1.

**Table 1:** Accessions used in this study.

**Species**	**Common name**	**Voucher**	**Origin**
*Papaver rhoeas*	Poppy	5194	JKIP KIT
*Mentha spicata* var. *crispa*	Spearmint	5391	WEL-Project; JKIP KIT
*Mentha x piperita*	Peppermint	5393	JKIP KIT
*Mentha aquatica*	Watermint	8680	JKIP KIT
*Lepidium sativum*	Cress	-	Rapunzel
*Arabidopsis thaliana* (Col 0)	Thale Cress	-	JKIP KIT
*A. thaliana* (Col 0) 35S::TuB6::GFP	Thale Cress	-	Nakamura *et al.* [58]

**Table 2:** Single compounds used in this study.

**Compound**	**CAS number**	**Purity [%]**	**Company**	**Catalogue number**
(−)-carvone	6485-40-1	analytical standard	Sigma-Aldrich	22 060
(−)-limonene	5989-54-8	analytical standard	Sigma-Aldrich	62 128
(+)-menthofuran	17 957–94-7	analytical standard	Sigma-Aldrich	63 661
*n*-hexane	110–54-3	hypergrade for organic trace analysis SupraSolv	Sigma-Aldrich	1 043 691 000

### Extraction of essential oils by hydro-distillation

To extract essential oil of each species, 50 g of fresh leaf material were excised, shock frozen and mildly squeezed in liquid nitrogen with mortar and pestle to break up the oil cavities of the glandular hairs and scales. The frozen powder was inserted into a still pot with a heating mantle and filled with distilled water to half of its height to ensure maximal surface. Water flow in the cooling system of the still was opposed to the direction of the still. After distilling at 100°C for 90 min, the extracted oil was quantified by a glass burette, harvested in small glass vials, and stored at 4°C for further analysis.

### GC–MS analysis

To get an insight into the chemical profiles of the different mint species a GC–MS analysis of the extracted oils was performed using an Agilent device equipped with a HP5 column (30 m, 0.25 mm I.D, 0.25 μm film thickness). For this, 10 mg of the essential oil was dissolved in 100 ml of hexane and 1 μl was injected. The GC injector was used in the split-less mode and set to a pressure of 16.7 psi and a temperature of 280°C. Mass spectra were collected using the electron impact (EI) mode at 70 eV, 33–450 m/z. Volatiles were eluted under the following conditions: 40°C (2 min isotherm), followed by heating at 10°C min^−1^ to 220°C and a subsequent step with accelerated heating at 30°C min^−1^ to 300°C, using helium (1.6 mL^.^min^−1^) as carrier gas. Data were analyzed using MassHunter Workstation software, Qualitative Analysis Navigator and Qualitative Analysis Workflows (version B.08.00, Agilent Technologies, Inc. 2016), identifying individual components by their chromatographic retention index (RI) and comparing the spectra to a library (Pal 600 K). The RIs were determined experimentally with a series of C7-C30 n-alkanes and compared with the values reported in the literature.

### Germination assays

To check the bioactivity of the main compounds of the essential oils (Tab. 2), several germination assays were conducted. First, a standard Cress germination test was performed to select possible candidates with high allelopathic effect using the protocol suggested by the International Seed Testing Association (www.seedtest.org). In brief, 50 Cress (*Lepidium sativum*) seeds were sown equidistantly on Whatmann filter paper (85 mm in diameter) wetted with 2 mL of distilled water in a Petri dish (90 mm x 16 mm). A coverslip (18 mm x 18 mm) with different volumes (0.1, 1, 10 μL corresponding to 1, 10 and 100 ppm, respectively) of the pure compounds, or the respective solvent *n-*hexane as control was placed in the center of the Petri dish, such that the compounds had to interfere with the germinating seeds through the gas phase mimicking natural conditions. After mounting, the Petri dishes were carefully sealed with Parafilm to prevent evaporation and cross-leakage of the gas phase between Petri dishes. The results were scored after incubation in darkness at 25°C for 3 or 5 days. The same experimental set-up was used for the germination assay with Poppy (*Papaver rhoeas*) except that the incubation took place with a 12 h light/12 h dark cycle at 20°C for 2 or 6 days. Lighting was provided by fluorescent tubes (TLD 36 W/25, Philips, Hamburg, Germany) with 120 μmol m^−2^·s^−1^ of photosynthetically available radiation. Since the germination rate in the controls was 100%, the inhibitory effect equaled the frequency of ungerminated seeds. Data represent means and standard deviations from three independent biological replications.

### Evans blue dye exclusion and FDA assay

To assess cellular effects of the different compounds, different strains of tobacco BY-2 (*Nicotiana tabacum* L. cv Bright Yellow-2) were used as suspension cultures [59]. In addition to a non-transformed cell line (BY-2 WT), a line expressing tobacco tubulin α3 with a N-terminal fusion of the GFP under control of a constitutive CaMV 35S promoter (TuA3) allows to follow microtubules *in vivo* [60], while the line GF11 expressing the second actin-binding domain of fimbrin (AtFim1) fused to GFP, also under the control of the CaMV 35S promotor, was employed to monitor actin filaments [61]. The cells were subcultured weekly by inoculating 1.5 mL of stationary cells into 30 mL of freshly prepared modified Murashige-Skoog (MS) medium [62] and cultivated at 26°C in darkness on an orbital shaker at 150 rpm. The transgenic lines were complemented with the respective antibiotics to maintain selective stringency (TuA3: 50 μg / mL Kanamycin; GF11: 30 μg/mL Hygromycin). Mortality was monitored using the Evans Blue Dye Exclusion Assay according to Gaff and Okong'o-Ogola [39]. Viability was examined using the fluorescence diacetate assay according to Finkbeiner *et al.* [63]. Aliquots of mitotically active cell suspension (200 μL, collected at day 3 after subcultivation) were transferred to a tube and incubated with 0.5% (v/v) of the respective compound, or the same concentration of the solvent *n-*hexane alone for 30 min. To copy the phenotype of the tubulin overexpression line TuA3 in the BY-2 WT, the WT cell line was incubated with 10 μM Taxol, a specific inhibitor of microtubule disassembly, for 1 h prior to treatment with the respective compound. Then, mortality was scored as described in Kühn *et al.* [64] and viability as described in Finkbeiner *et al.* [63]. Data represent means and standard errors from three independent biological replications with a population of 2500 individual cells per replication.

### Live-cell imaging

The response of the cytoskeleton to the compounds was followed *in vivo* using the microtubule-marker line TuA3 and the actin-marker line GF11 in mitotically active cell cultures, collected at day 3 after subcultivation. The compounds were administered through the gas phase as described in Sarheed *et al.* [26] and individual cells were followed over time by spinning disc confocal microscopy. In brief, 200 μl of the culture were transferred into a sterile tube under the clean bench and diluted with BY-2 medium to a proper density that allowed for good observation. An aliquot of 20 μl of this suspension was then transferred on a slide, and 0.5 μl of the test compound (or the solvent control *n-*hexane) were positioned on both sides of the cell suspension, before a 40 × 24 mm coverslip was placed on the slide. The distance of the test compounds was chosen such that they were close to the cells but remained separated by a gap. Thus, the compounds had to act through the gas phase. The cells were visualized with the Zeiss Observer.Z1 Spinning Disc microscope (Zeiss, Jena, Germany), using a 63x oil immersion objective upon excitation with blue light (488 nm) from an Argon-Krypton-laser (Zeiss, Jena, Germany) to detect the GFP signal. Individual cells were selected and followed for 35 minutes.

### Manufacturing of the microfluidic Root Chip

**Figure 11:**
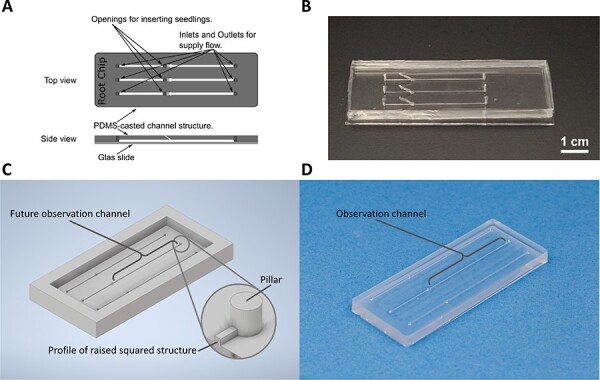
Design and fabrication of the Root Chip. **(A)** Design of the chip with three parallel channels equipped with inlets and outlets for supply flow and entry points for the roots. The channels are covered by a glass slide. (**B)** A specimen of the Root Chip ready for use. (**C)** Design of the mould for casting. (**D)** A PDMS cast generated by the mould in (C) as intermediate step of fabrication.

**Design of the Root Chip.** The Root Chip for this study was designed with focus on the functionalities to conveniently integrate the seedlings, and to preserve the physiology of the root over a long period (Fig. 11A). As a strategy we used a cast made from Polydimethylsiloxane (PDMS) covered by a thin coverslip made from glass. Since roots were observed through an inverse microscope, the channels had to be placed upside down on the coverslip.

The cast contains three straight channels with a squared cross section of 200 μm times 200 μm. Each channel has an inlet for the pregrown seedlings such that the root can enter the channel at an angle of 45°. In addition, in- and outlet allow to connect to tubing for providing the inserted root with nutritional media. To enable observation over several days, the length of the channel is 34 mm from the root-entry point. To avoid that seedling and tubing interfered with observation, the respective openings had to be placed at the bottom of the channel, opposite of the coverslip.

**Manufacturing.** To fabricate the Root Chip (Fig. 11B) by PDMS-casting, a mould is needed, prepared by micro-milling the complementary structure into Polymethyl methacrylate (PMMA), a transparent thermoplastic with good milling properties. Since the mould must be a mirror image of the intended PDMS cast, the channels correspond to straight ridges with a square cross section of 200 μm in length, while pillars mark the prospective inlets and outlets (Fig. 11C). This mould allows for a precise and time-efficient reproduction of the microstructures through a casting process. For casting itself the *SYLGARD™ 184 Silicone Elastomer Kit* (Dow, Midland, MI, USA) was employed according to the protocol of the manufacturer. To avoid the formation of air bubbles that would hamper observation and lead to disturbing cavities in the cast, air was removed thoroughly from the mixed PDMS under vacuum for 20 minutes prior and subsequent to the pouring step. The casted PDMS was then cured in an oven at 65°C for 1 hour and allowed to cool to 20°C before carefully demoulding the PDMS cast. Subsequently, the casts for in- and outlet were opened by a fine driller (diameter 1 mm). Furthermore, the inlets for seedling’s entry into the Root Chip were drilled out as well, but here at an angle of 45° to enable a smooth entry of the roots into the observation channels (Fig. 11D).

The Root Chip was then assembled in a clean room. First, the surfaces of the cast and the glass coverslip (Ref 302 980, Knittel Glass, Braunschweig, Germany) were thoroughly cleaned in an ultrasonic bath using a three-step process of acetone, isopropanol and de-ionized water for the coverslip and a two-step process of isopropanol and de-ionized water for the PDMS-cast. Then, both, the coverslip, and the PDMS-cast, were dried using compressed N_2_. The two components were then placed in a 4TEC plasma etcher (4-TEC Vakuum-Anlagenbau, Vierkirchen, Germany) with the bonding surface facing upwards and the surfaces were activated with a 100 W oxygen plasma for 7 s. Upon removing the two parts of the Root Chip from the etching chamber, cover slip and PDMS-cast were aligned manually and carefully pressed together to achieve the final bond.

For quality check, the fabricated chip was subjected to leak testing by pumping isopropanol through each individual channel at a flow rate of 500 μl^.^min^−1^ by means of a Syringe Pump (PHD ULTRA, Harvard Apparatus, Holliston, MA, USA). Only chips that were found tight under these extreme conditions were then dried, packaged, and used for the experiment.

### Analysis of *Arabidopsis* roots in the microfluidic Root Chip

**Preparation of *A. thaliana* seedlings.** Sterile seeds were sown into cut 200 μL micropipette tips (the seed was located at 10 mm from the pointed end), filled with 12 μL of ½ Murashige Skoog (MS, M5519, Sigma-Aldrich) medium containing 0.5 w/v % sucrose and 1 w/v % agar (05040, Millipore). After stratification at 4°C for 48 h in the dark, seedlings were grown under long day conditions (16 h light, 8 h dark) using white light from fluorescent bulbs (OSRAM L58W/956) with 70 μmol m^−2^·s^−1^ photosynthetically available radiation at 21°C for 5 days. For the measurement of root growth, the seeds were raised in the same manner, with the difference that they were sown equidistantly on filter paper strips placed on the agar in rectangular Petri dishes that were then placed vertical, such that the root was growing downwards. The effect of the compounds on growth was measured in *A. thaliana* ecotype Columbia 0, the response of microtubules was followed in line GFP-TuB6 [58].

**Integration into the microfluidic Root Chip.** The micropipette tips with the growing seedlings were stuck into the entry points of the Root Chip (see above) under a sterile hood and placed in the growth chamber for 3–4 days under a constant flow rate from base to tip of 8 μL min^−1^ of ½ MS medium supplemented with 0.5% sucrose supplied by a syringe pump (Fusion 200, Chemyx Inc., Stafford, TX, USA) connected to the device. The different compounds and *n*-hexane as solvent control were first diluted into a small volume of ethanol 96% (50 μL) and then adjusted to the final concentration with ½ MS medium containing 0.5% sucrose.

**Life imaging by confocal microscopy.** Compounds were administered at 1–4 μL^.^mL^−1^ (again in ½ MS medium containing 0.5% sucrose) to be able to observe acute effects on cell biology. To label the cell walls, propidium iodide was added to 2 μg^.^mL^−1^ [40]. The microfluidic device was subjected to a constant flow rate of the compound or control solutions and either microtubule or actin filament arrays of the root tip cells were visualized by confocal microscopy. To assess the validity of the chip system, control experiments were conducted, where root tip cells were observed directly placing the seedlings (at day 5 to 10 after germination) and the compound solutions between 22 x 32 mm coverslips and microscope slides. Confocal images were recorded with a Zeiss LSM 700 microscope (Zeiss, Jena, Germany) equipped with a 40x/1.3 NA oil immersion objective. The excitation and emission wavelengths for GFP were 488 nm and 510 nm, respectively. For propidium iodide labelling, the excitation and emission wavelengths were 555 nm and 617 nm, respectively. Observations were performed in the multi-tracking mode using 488 and 555 nm laser excitations. Root tips with a high fluorescence were selected and followed over time.

**Measurement of root growth.** To quantify the effect on root growth, the respective compound was mixed into the agar in a rectangular Petri dish at a final concentration of 0.1 μL^.^mL^−1^ (0.1 ppm) or 1 μL^.^mL^−1^ (1 ppm) with ½ MS medium containing 0.5% sucrose, and the paper strips with the germinated seedlings were then transferred to the test agar. The dish was then oriented vertically, such that the root was allowed to grow downwards. The Petri dishes with the root were then imaged daily over the following 5 days, and determining the length increment was measured using the segmented line measure tool plugin of ImageJ (imagej.nih.gov/ij). Data represent means and standard errors from two independent series of experiments (separate Petri dishes) with 15–22 individual roots per Petri dish. Samples were tested for normality by the Shapiro test. Since some samples were found to be deviate from a Gaussian distribution, statistical difference between controls and treated samples were probed using the non-parametric Wilcox test.

## Acknowledgements

The authors thank Mrs. Anna-Luise Kuppinger, JKIP Experimental Station of the Karlsruhe Institute of Technology, Karlsruhe, Germany, for the cultivation of the plant material and Dr. Kateřina Schwarzerová, Institute of Plant Physiology, Charles University, Prague, Czech Republic, for the provision of the *A. thaliana* (Col 0) 35S::TuB6::GFP marker line. NH’s research was supported by a Scholarship from the Graduate Funding from the German States (LGF). The microfluidic bioreactor was developed as part of a cooperation project funded by the German Federal Ministry of Education and Science (031B0065B). The study on microtubule responses in planta was supported by the European Fund for Regional Development (INTERREG V Upper Rhine program, DialogProTec). We gratefully acknowledge the support of the KIT-Publication Fund of the Karlsruhe Institute of Technology.

## Author contributions

N.H., P.N., A.S.: conceptualization; N.H., P.N., A.S., L.S., R.A.: methodology; N.H., P.N.: formal analysis; N.H., L.C., A.S., E.H., M.F.: investigation; L.S., R.A., M.F.: resources; N.H.: data curation; N.H., P.N.: writing—original draft; N.H., P.N.: writing—review & editing; N.H., P.N.: visualization; N.H., P.N.: supervision; P.N., N.H.: funding acquisition.

## Data availability

The data underlying this article are available in the article.

## Conflict of interest statement

The authors declare no conflict of interest.

## Supplementary Data

Supplementary data is available at Horticulture Research online.

## Supplementary Material

Web_Material_uhae151
